# Acta Orthopaedica 90-year anniversary

**DOI:** 10.1080/17453674.2020.1763561

**Published:** 2020-05-14

**Authors:** Anders Rydholm

*Acta Orthopaedica*, a nonprofit, Open Access journal, was founded in 1930 and is owned by the Nordic Orthopaedic Federation (NOF). The NOF is the federation of the national orthopedic societies of Denmark, Estonia, Finland, Iceland, Lithuania, The Netherlands, Norway, and Sweden representing over 5,500 orthopedic surgeons. *Acta* enables the mission of NOF, i.e., dissemination of evidence-based medicine, education and research.

Approximately 600 manuscripts are submitted to *Acta* each year, and of these around 100 are published after editorial and outside/external peer review. Articles come from all over the world but submissions and publications from the NOF countries are most common. *Acta* publishes numerous arthroplasty register studies—the first registers (Knee 1975 and Hip 1979, were started in Sweden)—and radiostereometry investigations (developed for orthopedic purposes in Sweden in 1974). Several of these register and radiostereometry studies are highly cited: see the top-10 cited articles below. During recent years *Acta* has also published an increasing number of randomized clinical trials.

This issue of Acta celebrates our 90-year anniversary with this overview of *Acta’s* development during the last 20 years and a compilation of the 10 most cited research articles during the last 20 years, each of them accompanied by a Guest editorial written by invited experts.

### Acta 2000–2020

The Open Access (OA) movement started in the 1990s and gained momentum in the early 2000s when PubMed Central (PMC) and OA BioMed Central were launched. *Acta* soon realized that OA had come to stay and in 2005 started, first of orthopedic journals, OA publication without cost for authors and readers. At this time we also made all *Acta* articles published since start 1930 electronically accessible cost-free. When we introduced OA we changed the journal’s name from *Acta Orthopaedica Scandinavica* to *Acta Orthopaedica* to emphasize *Acta’s* international character.

We foresaw that with OA we would lose subscriptions and in 2018 we introduced an Article Processing Charge (APC), which is common to most other journals that offer OA publication. APC relates to articles emanating from outside NOF. All Nordic orthopedic surgeons are members of NOF and pay a subscription for *Acta*, which means that they pay no APC. At the same time, we also stopped the print edition and *Acta* became an electronic-only journal. Accepted articles are, however, still compiled into 6 annual issues (each comprising around 20 original articles) presented as an e-zine and distributed to all collective NOF member subscribers.

All *Acta* articles are, since 2005, distributed under the terms of the Creative Commons Attribution Non-commercial Licence. This means that *Acta* has no copyright on your article; you as an author, and others, are free to use it for all non-commercial purposes.

In 2009 *Acta* started online prepublication; soon after acceptance the article is available on PubMed (Epub ahead of print) eliminating the previous delay caused by the wait for the print publication. In the same year *Acta* also started voluntary non-anonymous reviewing (also, the names of authors are open to the reviewers); the reviewers are named and thanked in the published article. There is an option for reviewers to remain anonymous, which is, however, rarely used: almost all > 200 annual reviewers sign their reports. We have a strong feeling this open system has improved the quality of the reviews, an observation also made by other journals.

Inappropriate use of statistics in biomedicine (including misuse/misunderstanding of p-values) leading to false conclusions has become increasingly apparent and discussed during the 2000s. It has been named as one of the explanations for the so-called replication crisis within science. Already in 1993 *Acta* included an experienced biostatistician among its editors, since then scrutinizing statistics in all manuscripts. He has also written many Editorials on the proper use of statistical methods and helped numerous authors to improve their manuscripts, thus implying well-founded conclusions. Several authors have expressed their appreciation for this help.

In 2019 Acta decided to publish Registered Reports, i.e., the methods and proposed analyses of planned studies are published as a journal article after peer-review has confirmed a meaningful study applying appropriate methods. Once the study is completed—and adheres to the initially approved proposal—publication in *Acta* is guaranteed irrespective of the study outcome.

We also decided to consider for publication manuscripts previously uploaded to a non-commercial preprint server, such as MedRxiv. We do not consider posting on a preprint server to be duplicate publication and this will not jeopardize consideration for publication in Acta after the ordinary review process.

#### Topics to be considered during 2020

- Make the review process transparent, i.e., make the complete preprint correspondence between authors, editors, and external reviewers electronically available together with the published article (a system already applied by some journals).
- Encourage publication of studies with (unexpected) null results (to avoid publication bias).
- Encourage publication of replication studies (cf. current biomedicine with a serious replication problem: the reproducibility crisis). There is a need for more systematic replication studies, regardless of their outcome. This applies also to clinical studies; in some way register studies of joint replacements are already close to replication studies—the same implants are studied in different populations managed at different hospitals.

### Top-10 articles 2000–2020

These 10 articles, published since 2000, were ranked (from 2,721 articles) by the fraction” Total number of cites/Years from publication”, to compensate for increasing possibilities of citation with time. The articles were cited around > 400–200 times. The abstract of each article is reproduced and each of them is accompanied by a Guest editorial written by invited experts. Acta thanks them for their excellent work.

Top-10 articles, 2000–2020 in descending order of number of citations

*Local infiltration analgesia: a technique for the control of acute postoperative pain following knee and hip surgery. A case study of 325 patients.* Dennis R Kerr and Lawrence Kohan. Acta Orthop 2008; 79(2): 174-83.

*Predictors of length of stay and patient satisfaction after hip and knee replacement surgery: Fast-track experience in 712 patients.* Henrik Husted, Gitte Holm, and Steffen Jacobsen. Acta Orthop 2008; 79(2): 168-73.

*Uncemented and cemented primary total hip arthroplasty in the Swedish Hip Arthroplasty Register: evaluation of 170,413 operations.* Nils P Hailer, Göran Garellick, and Johan Kärrholm. Acta Orthop 2010; 81(1): 34-41.

*Long-term registration has improved the quality of hip replacement: a review of the Swedish THR Register comparing 160,000 cases*. Peter Herberts and Henrik Malchau. Acta Orthop Scand 2000; 71(2): 111-21.

*Guidelines for standardization of radiostereometry (RSA) of implants.* Edward R Valstar, Richie Gill, Leif Ryd, Gunnar Flivik, Niclas Börlin, and Johan Kärrholm. Acta Orthop 2005; 76(4): 563-72.

*Why still in hospital after fast-track hip and knee arthroplasty?* Henrik Husted, Troels H Lunn, Anders Troelsen, Lissi Gaarn-Larsen, Billy B Kristensen, and Henrik Kehlet. Acta Orthop 2011; 82(6): 679-84.

*The epidemiology of proximal humeral fractures.* Charles M Court-Brown, Ashima Garg and Margaret M McQueen. Acta Orthop Scand 2001; 72(4): 365–71.

*The Norwegian Arthroplasty Register: 11 years and 73,000 arthroplasties*. Leif I Havelin, Lars B Engesaeter, Birgitte Espehaug, Ove Furnes, Stein A Lie, and Stein E Vollset. Acta Orthop Scand 2000; 71(4): 337-53.

*Effectiveness of hip or knee replacement surgery in terms of quality-adjusted life years and costs.* Pirjo Räsänen, Pekka Paavolainen, Harri Sintonen, Anna-Maija Koivisto, Marja Blom, Olli-Pekka Ryynänen, and Risto P Roine. Acta Orthop 2007; 78(1): 108-15.

*Patient satisfaction after knee arthroplasty: a report on 27,372 knees operated on between 1981 and 1995 in Sweden.* Otto Robertsson, Michael Dunbar, Thorbjörn Pehrsson, Kaj Knutson, and Lars Lidgren. Acta Orthop Scand 2000; 71(3): 262–7.

Anders Rydholm on behalf of all editors

